# Endocrine disruption: *In silico* perspectives of interactions of di-(2-ethylhexyl)phthalate and its five major metabolites with progesterone receptor

**DOI:** 10.1186/s12900-016-0066-4

**Published:** 2016-09-30

**Authors:** Ishfaq A. Sheikh, Muhammad Abu-Elmagd, Rola F. Turki, Ghazi A. Damanhouri, Mohd A. Beg, Mohammed Al-Qahtani

**Affiliations:** 1 King Fahd Medical Research Center, King Abdulaziz University, PO Box 80216, Jeddah, 21589 Kingdom of Saudi Arabia; 2 Centre of Excellence in Genomic Medicine Research, King Abdulaziz University, Jeddah, Kingdom of Saudi Arabia; 3 KACST Innovation Center in Personalized Medicine, King Abdulaziz University, Jeddah, Kingdom of Saudi Arabia; 4 Department of Obstetrics and Gynecology, King Abdulaziz University Hospital, Jeddah, Kingdom of Saudi Arabia

**Keywords:** Docking, Progesterone receptor, DEHP, 5-OH-MEHP, 5-oxo-MEHP, MEHP, 5-cx-MEPP, 2-cx-MMHP

## Abstract

**Background:**

Di-(2-ethylhexyl)phthalate (DEHP) is a common endocrine disrupting compound (EDC) present in the environment as a result of industrial activity and leaching from polyvinyl products. DEHP is used as a plasticizer in medical devices and many commercial and household items. Exposure occurs through inhalation, ingestion, and skin contact. DEHP is metabolized to a primary metabolite mono-(2-ethylhexyl)phthalate (MEHP) in the body, which is further metabolized to four major secondary metabolites, mono(2-ethyl-5-hydroxyhexyl)phthalate (5-OH-MEHP), mono(2-ethyl-5-oxyhexyl)phthalate (5-oxo-MEHP), mono(2-ethyl-5-carboxypentyl)phthalate (5-cx-MEPP) and mono[2-(carboxymethyl)hexyl]phthalate (2-cx-MMHP). DEHP and its metabolites are associated with developmental abnormalities and reproductive dysfunction within the human population. Progesterone receptor (PR) signaling is involved in important reproductive functions and is a potential target for endocrine disrupting activities of DEHP and its metabolites. This study used *in silico* approaches for structural binding analyses of DEHP and its five indicated major metabolites with PR.

**Methods:**

Protein Data bank was searched to retrieve the crystal structure of human PR (Id: 1SQN). PubChem database was used to obtain the structures of DEHP and its five metabolites. Docking was performed using Glide (Schrodinger) Induced Fit Docking module.

**Results:**

DEHP and its metabolites interacted with 19-25 residues of PR with the majority of the interacting residues overlapping (82-95 % commonality) with the native bound ligand norethindrone (NET). DEHP and each of its five metabolites formed a hydrogen bonding interaction with residue Gln-725 of PR. The binding affinity was highest for NET followed by DEHP, 5-OH-MEHP, 5-oxo-MEHP, MEHP, 5-cx-MEPP, and 2-cx-MMHP.

**Conclusion:**

The high binding affinity of DEHP and its five major metabolites with PR as well as a high rate of overlap between PR interacting residues among DEHP and its metabolites and the native ligand, NET, suggested their disrupting potential in normal PR signaling, resulting in adverse reproductive effects.

## Background

The chemical industry contributes significantly to the prosperity and economic development of modern society. However, many chemical compounds that are discharged into the environment due to industrial activity and leaching from consumer products interfere with the physiological functions of the exposed human and animal populations and are referred to as endocrine disrupting compounds (EDCs) [1–3].

**Fig. 1:**
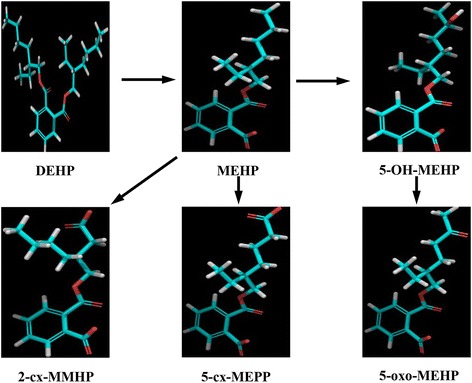
Two dimensional representation and a simplified pathway of di-(2-ethylhexyl)phthalate (DEHP) and its five major metabolites, mono-(2-ethylhexyl)phthalate (MEHP), mono-(2-ethyl-5-hydroxyhexyl)phthalate (5-OH-MEHP), mono-(2-ethyl-5-oxyhexyl)phthalate (5-oxo-MEHP), mono-(2-ethyl-5-carboxypentyl)phthalate (5-cx-MEPP), and mono-[2-(carboxymethyl)hexyl]phthalate (2-cx-MMHP)

Di-(2-ethylhexyl)phthalate (DEHP) is a high volume plasticizer used as a softener in polyvinyl chloride industry with a 54 % market share (2010 data) and is considered as one of the most common EDCs present in the environment [4]. DEHP is frequently used in the manufacture of medical devices, blood storage bags, surgical gloves, dialysis equipment, cosmetics, household and personal items such as soap, shampoo, detergents, adhesives, vinyl flooring, shower curtains, plastic bags, garden hoses, children’s toys, and many other plastic products [4]. Exposure of human population to DEHP occurs continuously through inhalation, ingestion, and skin contact [5]. Recently [6], DEHP was detected in 74 % of 72 common food items including infant foods, chicken, pork and other food items in a market in Albany, New York. DEHP is metabolized in the body by hydrolysis to a primary metabolite, mono-(2-ethylhexyl)phthalate (MEHP), which is then further metabolized into multiple hydroxylative and oxidative secondary metabolites [7, 8]. The four major secondary metabolites of DEHP are mono(2-ethyl-5-hydroxyhexyl)phthalate (5-OH-MEHP), mono(2-ethyl-5-oxyhexyl)phthalate (5-oxo-MEHP), mono(2-ethyl-5-carboxypentyl)phthalate (5-cx-MEPP) and mono[2-(carboxymethyl)hexyl]phthalate (2-cx-MMHP) [7, 8]. A simplified metabolic pathway of 5 major metabolites of DEHP is illustrated (Fig. 1).

DEHP and its metabolites have been detected in various human body fluids such as blood and breast milk [9], follicular fluid [10], amniotic fluid [11], cord blood of newborns [12] and urine [5] indicating immense potential for adverse health effects. Monoester metabolites rather than native DEHP are thought to be responsible for toxicity of DEHP [13] with secondary metabolites displaying a 100 fold increase in embryo-toxicity compared to MEHP [14]. In a recent study [15], positive associations were reported between total DEHP metabolites, MEHP, 5-OH-MEHP, and 5-oxo-MEHP levels in urine and plasma estradiol and ratio of estradiol to testosterone. Higher levels of MEHP, 5-OH-MEHP, and 5-oxo-MEHP were associated with lower sperm concentration, lower sperm motility, higher sperm apoptosis, and ROS generation [16]. Prenatal exposure with DEHP and its metabolites has been associated with reduced gestational age for pregnancies bearing male fetus [17], anogenital distance problems in male babies [18–20], cryptorchidism [21], altered reproductive hormone levels [22], hypospadias [23], intellectual and motor development in children [24], and preterm birth [25, 26]. Retrospective analyses of DEHP metabolites in pregnancy serum of mothers [27] indicated that prenatal exposure of children to DEHP was associated with reproductive problems during adolescence; higher 5-OH-MEHP level in prenatal maternal serum was related with lower semen volume and lower sperm concentrations and higher 5-cx-MEPP was associated with lower free testosterone concentrations.

Studies in rats and mice have also shown that exposure to DEHP can induce deleterious reproductive and endocrine effects [28–31]. In rats, prenatal DEHP treatment was associated with developmental abnormalities in male pups such as cryptorchidism, anogenital problems, and malformations of epididymis, vas deferens, seminal vesicles, prostate, and external genitalia collectively called as the phthalate syndrome, which is similar to effects of DEHP exposure in men [32, 33]. In vitro, MEHP and 5-OH-MEHP decreased gonocyte number and increased gonocyte apoptosis in rat testis organ culture [34].

In general, EDCs have been proposed to exert their toxic effects through interactions with nuclear steroid receptors, sex steroid binding proteins, and steroid enzymatic pathways regulating reproductive and endocrine functions [1]. Progesterone receptor (PR) belongs to the family of nuclear receptors and binds to progesterone, which is an important hormone involved in female reproductive function and maintenance of pregnancy [35, 36] as well as an important modulator of male reproductive function [37]. Interference in PR signaling leads to reproductive dysfunction and pregnancy failure [38]. Recently [39], docking studies of PR with three stereoisomers of DEHP have been reported. Docking of DEHP and its primary metabolite, MEHP, with PR have also been reported [40], however, the important secondary metabolites were not included in the study.

This study aimed at analyzing and comparing the structural binding characteristics of DEHP and its five major metabolites, MEHP, 5-OH-MEHP, 5-oxo-MEHP, 5-cx-MEPP, and 2-cx-MMHP with PR using *in silico* approaches. The study involved the delineation of the binding mechanism of all the six xeno-ligands with PR by molecular docking simulation and comparing the distinctive binding pattern and the interacting residues.

## Methods

### Data retrieval

The molecular structures of DEHP and its five major metabolites, MEHP, 5-OH-MEHP, 5-oxo-MEHP, 5-cx-MEPP, and 2-cx-MMHP were retrieved from PubChem compound database. The two dimensional structures of the ligands are illustrated (Fig. 1) and their abbreviations and PubChem compound identities (CIDs) are presented (Table 1). Schrodinger 2015 suite with Maestro 10.3 (graphical user interface) software (Schrodinger, LLC, New York, NY, 2015) was used for docking studies of DEHP and its five metabolites [39].

**Table 1:** Nomenclature, commonly used abbreviations, and PubChem IDs of di-(2-ethylhexyl)phthalate and its five major metabolites for docking study with human progesterone receptor (PR)

S.No.	Name	Abbreviation	PubChem ID
1	Di-(2-ethylhexyl)phthalate	DEHP	8343
2	Mono-(2-ethylhexyl)phthalate	MEHP	20393
3	Mono-(2-ethyl-5-hydroxyhexyl)phthalate	5-OH-MEHP	170295
4	Mono-(2-ethyl-5-oxyhexyl)phthalate	5-oxo-MEHP	119096
5	Mono-(2-ethyl-5-carboxypentyl)phthalate	5-cx-MEPP	149386
6	Mono-[2-(carboxymethyl)hexyl]phthalate	2-cx-MMHP	187353
7	Norethindrone	NET	6230

### Protein selection and preparation

The Protein Data Bank (PDB; http://www.rcsb.org/) was searched to retrieve the crystal structure of human PR (PDB code: 1SQN) with a resolution of 1.45 Å. The crystal structure was a co-complex with bound ligand, norethindrone (NET). The preparation of the co-complex crystal structure for docking analysis was done using protein preparation wizard workflow of Schrodinger Glide (Schrodinger suite 2015-3; Schrodinger, LLC) and was described in detail [39]. Briefly, the PDB structure was imported to docking software Glide and using protein preparation wizard workflow, OPLS-2005 force field, and Prime 3.0 module software water molecules were removed, hydrogen atoms and charges were added, and loops and missing side chains were built. The hydrogen bonding network was optimized and finally a geometry optimization was performed to a maximum root-mean-square deviation (RMSD) of 0.30 Å. For generating grid boxes, bound ligand (NET) in crystal complex was selected and used for docking of DEHP and its five metabolites.

### Ligand preparation, conformational search

The methodology described above [39] was employed to draw ligand structures (Fig. 1) using Maestro 10.3 (Maestro, version 10.3, Schrodinger, LLC). LigPrep module (Schrodinger 2015: LigPrep, version 3.1, Schrodinger, LLC) was used for preparation of ligands and correct molecular geometries and ionization at biological pH 7.4 were obtained by using the OPLS-2005 force field software.

### Induced fit docking

Schrodinger’s Induced Fit Docking (IFD) module was used for docking analyses of the DEHP and its five metabolites MEHP, 5-OH-MEHP, 5-oxo-MEHP, 5-cx-MEPP, and 2-cx-MMHP [39]. The ligands were submitted as starting geometries to IFD which is capable of sampling the minor changes in the backbone structure as well as robust conformational changes in side chains [41]. A softened-potential docking is performed in the first IFD stage where docking of the ligand occurs into an ensemble of the binding protein conformations. Subsequently, complex minimization for highest ranked pose is performed where both ligand and binding sites are free to move.

### Binding energy calculations

The ligand binding affinity calculations against the crystal complex was executed using Prime module of Schrodinger 2015 with MMGB-SA function.

## Results

**Fig. 2:**
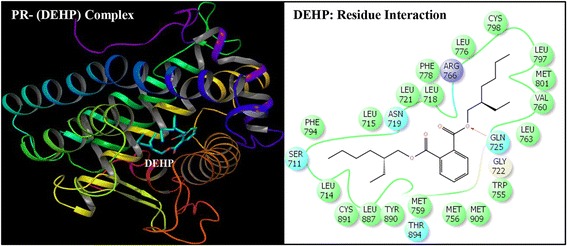
Ribbon form representation of docking complex of human progesterone receptor (PR) with di-(2-ethylhexyl)phthalate (DEHP) (left panel). Amino-acid residues in the binding pocket of PR involved in interactions with DEHP (right panel)

**Fig. 3:**
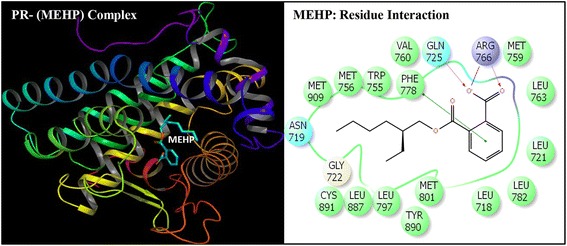
Ribbon form representation of docking complex of human progesterone receptor (PR) with mono-(2-ethylhexyl)phthalate (MEHP) (left panel). Amino-acid residues in the binding pocket of PR involved in interactions with MEHP (right panel)

**Fig. 4:**
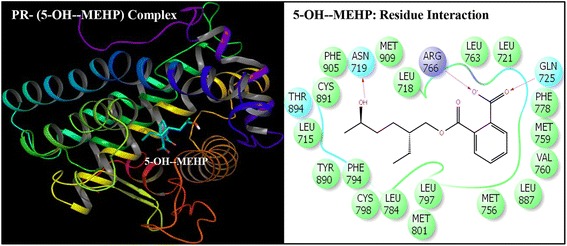
Ribbon form representation of docking complex of human progesterone receptor (PR) with mono-(2-ethyl-5-hydroxyhexyl)phthalate (5-OH-MEHP) (left panel). Amino-acid residues in the binding pocket of PR involved in interactions with 5-OH-MEHP (right panel)

**Fig. 5:**
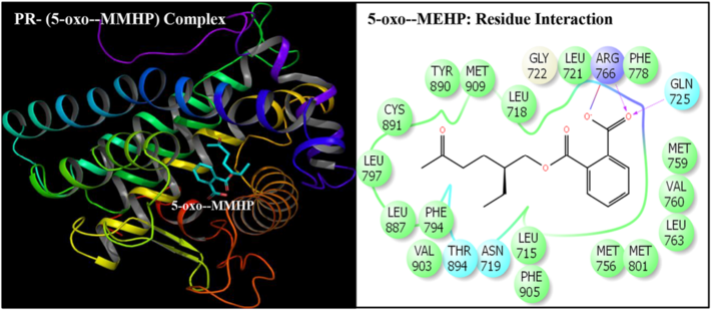
Ribbon form representation of docking complex of human progesterone receptor (PR) with mono-(2-ethyl-5-oxyhexyl)phthalate (5-oxo-MEHP) (left panel). Amino-acid residues in the binding pocket of PR involved in interactions with 5-oxo-MEHP (right panel)

**Fig. 6:**
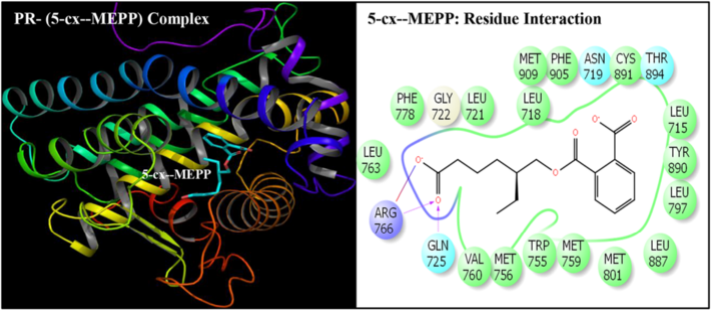
Ribbon form representation of docking complex of human progesterone receptor (PR) with mono-(2-ethyl-5-carboxypentyl)phthalate (5-cx-MEPP) (left panel). Amino-acid residues in the binding pocket of PR involved in interactions with 5-cx-MEPP (right panel)

**Fig. 7:**
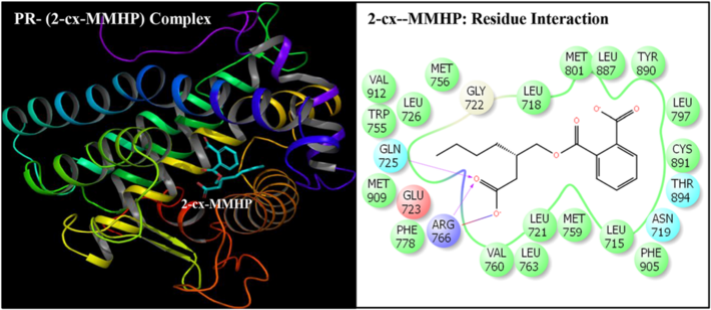
Ribbon form representation of docking complex of human progesterone receptor (PR) with mono-[2-(carboxymethyl)hexyl]phthalate (2-cx-MMHP)(left panel). Amino-acid residues in the binding pocket of PR involved in interactions with 2-cx-MMHP (right panel)

**Fig. 8:**
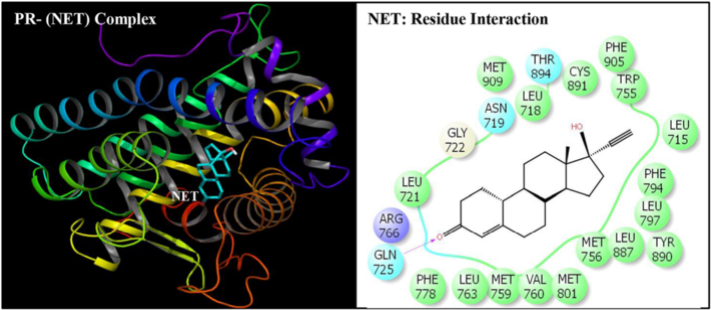
Ribbon form representation of docking complex of human progesterone receptor (PR) with native co-complex ligand norethindrone (NET) (left panel). Amino-acid residues in the binding pocket of PR involved in interactions with NET (right panel)

Successful execution of IFD for docking simulation of DEHP and its five major metabolites, MEHP, 5-OH-MEHP, 5-oxo-MEHP, 5-cx-MEPP, and 2-cx-MMHP against the ligand binding pocket of PR resulted in multiple docking poses for each ligand. The best pose for each ligand was analyzed further for *in silico* data considerations and the resulting data is presented here (Figs. 2, 3, 4, 5, 6 and 7). Similarly, for the co-complex bound ligand (NET) of PR the data for the best pose after IFD are illustrated (Fig. 8).

Docking complexes of DEHP and its five major metabolites, MEHP, 5-OH-MEHP, 5-oxo-MEHP, 5-cx-MEPP, and 2-cx-MMHP displayed interactions with 19-25 amino acid residues of PR (Figs. 2, 3, 4, 5, 6 and 7, Table 2). The bound ligand, NET, displayed interactions with 22 residues of PR in the NET-PR docking complex (Fig. 8; Table 2). DEHP and its five metabolites shared 18-21 PR interacting residues with the bound native PR ligand, NET, (commonality of 82-95 %; Table 2). For each of the native ligand, NET, and DEHP and its five metabolites, 16 PR interacting residues (Leu-718, Asn-719, Leu-721, Gln-725, Met-756, Met-759, Val-760, Leu-763, Arg-766, Phe-778, Leu-797, Met-801, Leu-887, Tyr-890, Cys-891, Met-909) were common (Table 3). The PR interacting residues, Leu-715 and Thr-894 were also common between bound ligand, NET, and DEHP and all of its metabolites except MEHP, and residue Gly-722 was common between NET and all ligands except 5-OH-MEHP (Table 3). In addition, two residues, Trp-755 and Phe-905 were common among NET and 4 of 6 ligand molecules (not shown). DEHP and each of its five metabolites and bound native ligand, NET, formed a hydrogen bonding interaction against residue Gln-725 of PR. In addition, MEHP, 5-oxo-MEHP, 5-cx-MEPP, and 2-cx-MMHP each formed two hydrogen bonding interactions with residue Arg-766 of PR. The metabolite 5-OH-MEHP formed only one hydrogen bonding interaction with Arg-766 but was also involved in a hydrogen bonding interaction with another residue, Asn-719, of PR. The IFD score, Dock score, and Glide score for all the docked xeno-ligands and bound native ligand, NET, are presented (Table 2). The binding affinity values (MMGB-SA values) were highest for NET followed by DEHP, 5-OH-MEHP, 5-oxo-MEHP, MEHP, 5-cx-MEPP, and 2-cx-MMHP (Table 2).

**Table 2:** Number of interacting residues, number and percentage of residues common with native ligand norethindrone (NET), Induced Fit Docking (IFD) Score, Dock score, Glide score and binding affinity values (MMGB-SA values) of di-(2-ethylhexyl)phthalate (DEHP), mono-(2-ethylhexyl)phthalate (MEHP), mono-(2-ethyl-5-hydroxyhexyl)phthalate (5-OH-MEHP), mono-(2-ethyl-5-oxyhexyl)phthalate (5-oxo-MEHP), mono-(2-ethyl-5-carboxypentyl)phthalate (5-cx-MEPP), and mono-[2-(carboxymethyl)hexyl]phthalate (2-cx-MMHP) and native co-complex ligand, NET, after IDF with human progesterone receptor (PR)

S. no.	Ligand	Number of interacting residues	Number of interacting residues common with NET (%)	IFD score	Docking score (Kcal/mol)	Glide score (Kcal/mol)	MMGB-SA (Kcal/mol)
1	DEHP	25	20 (91 %)	-563.15	-9.59	-9.59	-131.26
2	MEHP	19	18 (82 %)	-560.47	-8.40	-8.40	-84.18
3	5-OH-MEHP	22	20 (91 %)	-561.24	-8.95	-8.95	-90.78
4	5-oxo-MEHP	22	20 (91 %)	-561.72	-8.83	-8.83	-87.24
5	5-cx-MEPP	21	21 (95 %)	-562.87	-10.52	-10.52	-80.01
6	2-cx-MMHP	24	21 (95 %)	-562.01	-9.02	-9.02	-68.59
7	NET	22	22 (100 %)	-566.25	-12.13	-12.13	-139.00

**Table 3:** Amino-acid residues of human progesterone receptor that were common among co-complex natural ligand, norethindrone (NET), and di-(2-ethylhexyl)phthalate (DEHP) and its five major metabolites, mono-(2-ethylhexyl)phthalate (MEHP), mono-(2-ethyl-5-hydroxyhexyl)phthalate (5-OH-MEHP), mono-(2-ethyl-5-oxyhexyl)phthalate (5-oxo-MEHP), mono-(2-ethyl-5-carboxypentyl)phthalate (5-cx-MEPP), and mono-[2-(carboxymethyl)hexyl]phthalate (2-cx-MMHP)

S. no	Interacting residue	S. no	Interacting residue
1	Leu-715^a^	12	Phe-778
2	Leu-718	13	Leu-797
3	Asn-719	14	Met-801
4	Leu-721	15	Leu-887
5	Gly-722^b^	16	Tyr-890
6	Gln-725	17	Cys-891
7	Met-756	18	Thr-894^a^
8	Met-759	19	Met-909
9	Val-760		
10	Leu-763		
11	Arg-766		

Amino-acid residues indicated by superscript ^a^ were not shared by MEHP and the residue indicated by superscript ^b^ was not shared by 5-OH-MEHP

## Discussion

Di-(2-ethylhexyl)phthalate (DEHP) is a widely used phthalate compound representing more than half of all phthalate compounds manufactured worldwide for use in the industry as a plasticizer. Several reviews showed that DEHP is a universally prevalent environmental contaminant and behaves as a reproductive and developmental toxin [5, 28, 29, 32, 42]. Several epidemiological reports have identified DEHP and its metabolites as the cause of adverse effects on various systems of the body including endocrine and reproductive system [28, 29, 32]. Many studies have reported developmental problems during prenatal period and postnatal period in unborn and new born children as a result of gestational exposure of mothers to DEHP [18–20, 24, 42, 43]. In women, higher urinary or serum levels of DEHP and its metabolites were associated with problems in conception, endometriosis, and high rates of miscarriage, delayed or preterm gestation, and pregnancy associated toxemia and preeclampsia [25, 26, 28, 29]. In men, higher urinary or serum levels of DEHP and its metabolites were linked with lower semen volume, lower sperm concentrations, lower sperm motility, higher sperm apoptosis, and lower testosterone concentrations [5, 16, 27, 32, 44, 45]. Due to side effects of DEHP mentioned above, it has been banned since 2009 in the United States for use in children’s toys and the European Union has also classified DEHP as a reproductive toxicant. However, DEHP continues to be manufactured and used in many countries across the world.

DEHP is metabolized in the body by hydroxylative and oxidative reactions to many metabolic products which include five major metabolites: MEHP, 5-OH-MEHP, 5-oxo-MEHP, 5-cx-MEPP, and 2-cx-MMHP ([7, 8]; see Introduction section). The toxicity of DEHP in the body is attributed mainly to the actions of its secondary metabolites [13, 14]. Progesterone receptor signaling is an essential pathway controlling reproductive function and is involved in reproductive periodicity and establishment and maintenance of pregnancy [35, 36]. DEHP and the indicated five major metabolites can act as potential xeno-ligands for PR and disrupt the normal progesterone signaling pathway and this could be one of the important mechanisms which lead to adverse effects in the human population. In the present study, docking simulations of DEHP and its five major metabolites namely, MEHP, 5-OH-MEHP, 5-oxo-MEHP, 5-cx-MEPP, and 2-cx-MMHP were performed with PR and comparison of docking displays and interacting residues was performed among the ligands and the co-complex bound native ligand, norethindrone (NET) of PR crystal structure.

Induced Fit Docking of DEHP and its five metabolites with PR showed that all the six xeno-ligands fitted well into the steroid binding pocket of the receptor. The high binding affinity values, IFD scores, and dock scores indicated that the docking complexes formed by DEHP, MEHP, 5-OH-MEHP, 5-oxo-MEHP, 5-cx-MEPP, and 2-cx-MMHP with PR were in their most favorable conformation. A number of important PR amino acid residues interacted through hydrophobic and hydrogen-bonding interfaces with each of the six xeno-ligands during docking simulation contributing to the ligand-PR docking complex stability. A consistent and high overlapping (82-95 % commonality) of the interacting residues of PR among native bound ligand, NET, and DEHP and its metabolites suggested a common platform of action. This was further supported by the fact that 16 of the 22 PR residues interacting with bound native ligand, NET, also interacted with DEHP and each of the five metabolites. In addition, DEHP and each of its five metabolites, and bound native ligand, NET, formed a hydrogen bonding interaction against residue Gln-725 of PR altogether pointing to the common structural binding characteristics of the native bound ligand and the six xeno-ligands. Commonality of structural binding characteristics of bound native ligand, NET, and DEHP and its metabolites with PR suggest, on a preliminary basis, potential disruption of PR function by DEHP and its metabolites.

To the best of our knowledge, the current study is the first structure based report for docking stimulation of secondary metabolites of DEHP with PR. In vitro competitive binding of DEHP and its metabolites with PR are seemingly unavailable. Docking studies of PR with three stereoisomers of DEHP have recently been reported [39]. Docking of DEHP and its primary metabolite, MEHP, with PR have also been reported [40]. The results of the current study with docking of DEHP and PR support the results of the reported study [40] showing residues Gln-725, Arg-766 and Phe-778 as the crucial interacting residues of PR interaction with DEHP. The importance of the current study lies in the fact that the secondary metabolites of DEHP viz. 5-OH-MEHP, 5-oxo-MEHP, 5-cx-MEPP, and 2-cx-MMHP are the best biomonitoring markers of DEHP in the urine or blood and are potentially more potent disruptors because of their long elimination half-life compared to the primary metabolite, MEHP [7, 8]. Approximately 75 % of a single dose of DEHP was excreted in urine within two days; 67 % was excreted within the first 24 h which included 6 % MEHP, 23 % 5-OH-MEHP, 15 % 5-oxo-MEHP, 19 % 5-cx-MEPP, and 4 % 2-cx-MMHP (Koch et al. [8]). Of the 3.8 % excreted in the next 24 h, more than 75 % included 5-cx-MEPP and 2-cx-MMHP and the rest included 5-OH-MEHP and 5-oxo-MEHP indicating long elimination half-lives of the former two secondary metabolites.

Although not related to the progesterone receptor, DEHP treatment inhibited progesterone secretion from human luteal cells in culture [46]. Furthermore, in vivo treatment of DEHP decreased secretion of progesterone in mice [47] and in vitro treatment of MA-10 mouse Leydig cells with MEHP resulted in inhibition of steroidogenesis including progesterone secretion [48]. Interestingly, in sheep, DEHP causes shortening of estrous cycles due to a reduction in the size and lifespan of CL, however, in contrast to mice, an increase in circulating concentrations of progesterone was noted [49]. Conversely, MEHP treatment was associated with an increase in steroidogenesis including progesterone concentrations in cultured rat ovarian follicles [50]. Apparently, direct studies involving treatments with secondary metabolite compounds namely 5-OH-MEHP, 5-oxo-MEHP, 5-cx-MEPP, and 2-cx-MMHP in laboratory animals or in in vitro cell cultures are not available. It goes without saying that no single mechanism or pathway can explain the endocrine disrupting effects of DEHP and its metabolites on reproductive and endocrine systems in the human body. As an example, PPAR alpha was thought to be a possible pathway of adverse effects of DEHP in mice, however, the toxic effects were observed despite the use of PPAR alpha null mice suggesting the involvement of additional pathways [51]. Besides the PR signaling pathway, multiple other pathways could mediate the adverse effects of DEHP and its metabolites in the body. Androgen receptor pathway could also be an important mechanism as agonistic (androgenic) and antagonistic (antiandrogenic) actions of DEHP and other phthalate compounds have been shown at the androgen receptor level [52]. This study showed that DEHP and all its five major metabolites were able to bind to PR with structural binding characteristics that were common with the bound native ligand, NET, of PR. Hence, DEHP and its five metabolites have potential to interfere with the binding of progesterone to its receptor resulting in adverse effects and the dysfunction of progesterone signaling.

## Conclusion

This study was undertaken to understand the structural binding mechanisms of DEHP and its five major metabolites (MEHP, 5-OH-MEHP, 5-oxo-MEHP, 5-cx-MEPP, and 2-cx-MMHP) with PR in order to predict their potential adverse effects on progesterone signaling. The results indicated, a high percentage of overlap (82-95 %) among the interacting residues of PR for the native bound ligand, NET, and for DEHP and its metabolites. The structural binding similarities were further supported by a common hydrogen bonding interaction between Gln-725 residue of PR and DEHP,each of its five metabolites, and bound native ligand, NET. Therefore, on a preliminary basis, the six xeno-ligands have potential disruptive activities in the binding of progesterone to its receptor resulting in the dysfunction of progesterone signaling and adverse effects.

## Abbreviations

2-cx-MMHP: mono[2-(carboxymethyl)hexyl]phthalate
5-cx-MEPP: mono(2-ethyl-5-carboxypentyl)phthalate
5-OH-MEHP: mono(2-ethyl-5-hydroxyhexyl)phthalate
5-oxo-MEHP: mono(2-ethyl-5-oxyhexyl)phthalate
DEHP: Di-(2-ethylhexyl)phthalate
EDC: Endocrine disrupting compound
IFD: Induced fit docking
MEHP: mono-(2-ethylhexyl)phthalate
PR: Progesterone receptor
RMSD: Root-mean-square deviation
WHO: World Health Organization
